# HIV Eradication: Combinatorial Approaches to Activate Latent Viruses

**DOI:** 10.3390/v6114581

**Published:** 2014-11-21

**Authors:** Elisa De Crignis, Tokameh Mahmoudi

**Affiliations:** Department of Biochemistry, Erasmus University Medical Center, Rotterdam 3015 CN, The Netherlands; E-Mail: e.decrignis@erasmusmc.nl

**Keywords:** HIV latency, latency reversing agents, shock and kill strategy, HIV transcription regulators

## Abstract

The concept of eradication of the Human Immune Deficiency Virus (HIV) from infected patients has gained much attention in the last few years. While combination Anti-Retroviral Therapy (c-ART) has been extremely effective in suppressing viral replication, it is not curative. This is due to the presence of a reservoir of latent HIV infected cells, which persist in the presence of c-ART. Recently, pharmaceutical approaches have focused on the development of molecules able to induce HIV-1 replication from latently infected cells in order to render them susceptible to viral cytopathic effects and host immune responses. Alternative pathways and transcription complexes function to regulate the activity of the HIV promoter and might serve as molecular targets for compounds to activate latent HIV. A combined therapy coupling various depressors and activators will likely be the most effective in promoting HIV replication while avoiding pleiotropic effects at the cellular level. Moreover, in light of differences among HIV subtypes and variability in integration sites, the combination of multiple agents targeting multiple pathways will increase likelihood of therapeutic effectiveness and prevent mutational escape. This review provides an overview of the mechanisms that can be targeted to induce HIV activation focusing on potential combinatorial approaches.

## 1. Introduction

Since its discovery in 1983, the Human Immunodeficiency Virus (HIV) has sustained one of the major pandemics in the history of mankind. Hopes for the achievement of a cure for HIV infection were raised in the late 1990s, after the introduction of Combined Antiretroviral Therapy (c-ART). c-ART showed to be effective in controlling viral replication and dramatically decreased AIDS-related morbidity and mortality. Following this initial success, early mathematical models based on the viral load decay rate in presence of c-ART estimated that eradication could be reached after 2–3 years of suppressive therapy [1]. However, it was soon evident that a pool of replication competent viruses persists in patients despite therapy, leading to viral rebound upon treatment interruption [2,3,4,5]. Thus, to control the infection, HIV-1 infected patients must take lifelong medication, turning HIV infection into a chronic disease [6]. The chronic aspects of the disease have important consequences on life expectancy and management of HIV patients. Lifelong therapy has adverse effects and does not completely restore the immune system of infected patients, which maintains higher rates of morbidity and mortality compared to healthy individuals. Moreover, from a public health care perspective, providing lifelong therapy and health care support for all infected individuals poses a great economic challenge [7]. Considering these limitations, a significant effort has been recently put into finding new strategies to eradicate the virus or allow the patients to control viral replication in absence of therapy.

## 2. Proof of Concept for a Functional Cure

The use of fully suppressive c-ART has led to important insights into the nature and dynamics of the persisting latent viral pool. In c-ART treated patients, HIV viral load in peripheral blood progressively decreases over time to undetectable levels using commonly used diagnostic assays. Since the free virus has a very short half-life [8] and c-ART effectively blocks new rounds of infection, in treated individuals the decay rate of viremia depends on the lifespan of infected cells [1]. Death of infected CD4 T lymphoblasts caused by viral cytopathic effects or host immune responses is responsible for the first phase of viral load decay, that can be observed within 2 weeks following start of therapy [9]. Macrophages, dendritic cells and partially activated CD4s, which are thought to be other infected cell sources producing HIV, have longer half-lives and around 4–6 weeks of c-ART is required for their elimination. This period corresponds to the second phase of decay. In the majority of patients, this phase is followed by the decrease of plasma viral load below the limit of detection. The third phase of decay is slower, and it is estimated that nearly 70 years of effective c-ART will be necessary to eradicate HIV from infected patients [9]. Because of their extended half-life (44 months) and self-renewal capacity due to homeostatic proliferation [4,10,11], CD4 T cells represent the major compartment involved in HIV persistence. Two possible mechanisms can account for the long term persistence of HIV in this phase: an ongoing low-level replication that continuously replenishes the viral reservoir or the presence of a completely silent latent reservoir that can be occasionally reactivated [12]. However, effects of therapy intensification trials on residual viremia [13,14] proved to be inconsistent. In addition, studies indicate the absence of phylogenetic evolution of HIV during and after years of antiretroviral therapy [15,16]. These observations are consistent with the latter hypothesis and support the notion that the existence of a pool of quiescent latently infected cells is the major source of viral persistence in c-ART treated suppressed patients and a barrier to a cure for HIV.

Recently, several cases have fueled a renewed effort in finding a cure for HIV-1 infection. In the first case, T.R. Brown (the “Berlin patient”) who developed acute myelogeneous leukemia, was treated with an allogenic hematopoietic stem cells transplant (HSCT) from a donor harboring a mutated form of the CCR5 receptor that confers resistance to infection with CCR5-tropic strains. After undergoing the transplant in 2007, the patient stopped antiretroviral therapy and did not show any sign of rebounding infection [16,17]. A strong reduction of the reservoir size was also observed in two patients who underwent HSCT from a wild-type CCR5 donor. Following transplantation, the patients remained on c-ART for 2 or more years and showed undetectable levels of HIV RNA, DNA or replication-competent virus both in peripheral blood and gut biopsies [18]. Given that the virus remained undetectable for multiple years, c-ART was interrupted. However, patients experienced viral rebound after 12 and 32 weeks, respectively, and developed symptoms consistent with an acute infection process involving the transplanted cells. Interestingly, in both cases, phylogenetic analysis demonstrated that viral rebound was initiated by one or few latent proviruses consistent with the persistence of a minimal reservoir of infected cells despite HSCT [19].

The second case, reported in 2013, is that of a child born from an HIV-infected mother not in therapy that received antiretroviral drugs within the first 30 h postpartum and was treated for the first 15 months before therapy interruption [20]. The child did not exhibit signs of viral replication for two years after treatment interruption, generating hopes that this would be the second case of cure. Despite the recent report of viral rebound in the child [21], this case strongly supports the notion that early c-ART can effectively limit the establishment of the reservoir thereby delaying the onset of the disease. A similar strategy, limiting the seeding of the reservoir by initiating therapy during acute infection, proved to be promising also in adult patients. Recent clinical studies demonstrated that treatment of patients in the acute stage not only decreased the levels of cell-associated DNA and the overall size of the latent reservoir, but also reduced the proportion of proviruses present in Central Memory T cells, which constitute the most long lived population of CD4 T cells [22]. Studies aimed at evaluating the efficacy of immediate treatment have identified HIV patients, defined as post treatment controllers, who are able to control viremia for several years after therapy interruption [23,24,25,26]. In the ARNS VISCONTI study, the 14 post treatment controllers displayed a comparable genetic background and immune response to chronic patients rather than elite controllers. Thus, in these patients the ability to control HIV replication in absence of therapy is attributed to c-ART initiation in the early phase of infection and prolonged viral suppression [27].

Taken together, these cases constitute the proof-of-concept that a cure is achievable in HIV patients, either constituting the complete eradication of the virus (sterilizing cure), or the control of viral load in the absence of c-ART for prolonged periods of time (functional cure). In both cases, the fundamental step for the achievement of cure is a strong reduction of the size or complete eradication of the latent reservoir.

Since the majority of HIV patients are diagnosed during the chronic phase of the infection, when a large reservoir of latently infected cells is already established, the first goal to obtain a functional cure is to reduce the size of the existing reservoir. Using a stochastic mathematical model, Hill and coworkers recently estimated that a reduction of 1000–3000 fold of the reservoir size will be necessary to allow 1 year treatment interruption without rebound. According to this model, achieving complete HIV eradication with no risk of viral rebound in the majority of patients will require a minimum 4.8 logs reduction of the reservoir size [28]. In chronic patients, this reduction in the latent reservoir could theoretically be achieved using therapeutics to induce viral replication from latency. Infected cells harboring activated HIV would then be exposed to immune clearance and subject to viral cytopathic effects, leading to depletion of the latently infected cells that constitute the reservoir [29,30]. The presence of intensified c-ART during reactivation treatment would prevent new rounds of infection by replication-competent viruses. This strategy requires two steps: the identification of molecules able to stimulate HIV transcription and translation from a large proportion of latently infected cells and the boosting of the immune system in order to promote the elimination of cells harboring reactivated HIV.

## 3. Latency: The Consequence of a Block in HIV Gene Expression

Retrotranscription and integration into the host genome are key steps in the HIV viral cycle. Following integration, preferentially into actively transcribed genes [31], the HIV genome, similar to an endogenous cellular gene, becomes subject to a complex network of molecular mechanisms that determine and regulate its expression. The expression of the HIV genome is controlled by the viral promoter or 5' Long Terminal Repeat (LTR), whose transcription is critically dependent on the availability of host cell transcription factors and their associated cofactors and can lead either to the establishment of a productive infection or entry into a repressed latent state.

### 3.1. First Block: Transcription Initiation

Latent proviruses are molecularly characterized by a compacted repressive highly conserved chromatin structure. At the 5' LTR, the proviral promoter in its silent state is organized into 2 nucleosomes, Nuc-0 (−415/−255), Nuc-1 (+10/+155) and DHS-1 (−255/+10), an intervening DNA region hypersensitive to digestion with nucleases and restriction enzymes [32]. This latter region with absent or poorly positioned nucleosomes presents binding sites for multiple host transcriptional regulators [33].

The presence of Nuc-0, upstream of the modulatory region, and Nuc-1, flanking the core promoter, is the hallmark of a repressed 5' LTR. Nuc-1 is highly repressive to transcription and becomes rapidly and specifically disrupted upon activation [34,35,36]. This strictly defined repressive nucleosomal structure is accomplished via at least three epigenetic molecular mechanisms, which the cell has co-evolved to regulate gene expression: 1. The activity of chromatin remodeling complexes that use energy from ATP hydrolysis to actively position or remodel nucleosomes, 2. The posttranslational modifications of histones, including acetylation and methylation to modulate nucleosomal structure, and 3. DNA methylation. As detailed below all three mechanisms have been shown to play a role in regulation of the latent HIV promoter structure and as such represent attractive molecular targets in latent HIV re-activation efforts.

Scattered throughout the HIV LTR are consensus binding sites for a number of cellular transcriptional activators, including NF-κB, AP-1, NFAT, LEF1 and SP-1 [33,34,37]. In resting CD4 T cells, which exist in a quiescent, metabolically inactive state, many of these factors are sequestered in the cytoplasm and are recruited to the nucleus only after cellular activation [38]. Additionally, a number of transcriptional repressors including YY-1, LSF, CbF-1, p50 homodimers have also been described to bind to the LTR and repress HIV transcription [33,39,40,41,42]. The concerted activity of these transcription activators and repressors, and their associated activating or repressive transcriptional cofactors, tightly controls expression of the HIV genome. The latent HIV reservoir is thought to be largely formed as a consequence of infection of partially activated CD4 T cells that revert to a resting state. During this process a number of activators which are essential for HIV expression, such as NF-κB and NFAT transcription factors, are sequestered into the cytoplasm, while the establishment of a more condensed chromatin landscape further contributes to repression of HIV transcription [38,43].

### 3.2. Second Block: Transcription Elongation; the Critical Role of Tat

Following chromatin remodeling and the binding of transcription factors, RNA pol II is positioned and poised on the transcription start site (TSS) of the HIV promoter. Under basal conditions, RNA Pol II pauses close to the TSS and produces only short transcripts due to the presence of the negative elongation factors NELF and DSIF [44,45,46,47]. To overcome this inhibition and progress through to production of full length transcripts, HIV encodes a strong transactivator, Tat. Tat, which specifically interacts with the nascent structured TAR RNA sequence located at the 5' HIV LTR, recruits the Super Elongation Complex (SEC) to the nascent HIV transcripts [48]. A key component of SEC is the positive transcription elongation complex (P-TEFb) which is comprised of cyclinT1 and the kinase CDK9 [49]. Once recruited to the HIV promoter, CDK9 phosphorylates NELF and the carboxy-terminal domain of RNA pol II, overcoming NELF and DSIF inhibition and increasing RNA pol II processivity [50,51]. Besides recruiting SEC, Tat, which is itself subject to extensive post-translational modifications [52] invites the docking and activity of additional transcriptional co-activators, such as acetyltransferases and ATP-dependent chromatin-remodeling complexes to activate transcription [48].

Due to its prominent role in transcription regulation, intracellular levels of P-TEFb are tightly controlled. Availability of P-TEFb relies on two mechanisms: its interaction with 7SK small nuclear ribonucleoprotein complex (snRNP) which sequesters P-TEFb in a metabolically inactive state and the intracellular levels and activity of its components CycT1 and CDK9. The 7K snRNP complex contains several proteins, including the nuclear proteins HEXIM1 or HEXIM2, which inhibit the kinase activity of CDK9. In actively replicating cells, the majority of P-TEFb is sequestered into the 7SK snRNP complex and Tat activity induces its release by competing with HEXIM for CycT1 binding [53].

In contrast, in resting CD4 T cells, the levels of 7SK snRNP are very low, thus the main P-TEFb regulatory mechanism in these cells is likely to be the posttranslational regulation of CycT1 levels [54,55,56]. In these cells, reactivation of HIV will require accumulation of CycT1, activation of CDK9, assembly into the 7SK snRNP complex and Tat mediated recruitment to the HIV LTR.

## 4. Cell Model Systems in the Study of HIV Latency

In order to devise strategies to reverse HIV latency it is essential to unravel the complex molecular nature of HIV latency and identify the key molecular mechanisms and players involved in maintenance of HIV latency and its re-activation. Critical to these investigations is the development and availability of suitable and efficient model systems that allow molecular examination of HIV latency. Latent HIV infected CD4 T cell and monocytic cell lines are widely used model systems that have proven to be immensely useful for studies aimed at delineating the molecular mechanisms that control HIV latency and transcription. These models include clonal cell lines that contain integrated minimal HIV-derived viruses harboring mutations in the HIV Tat-TAR axis, or its replacement by a Tet-on system resulting in low basal but inducible transcription [57,58,59,60]. Studies using these cell lines have generated much insight as to the molecular determinant of transcription at the HIV LTR. The J-Lat model system was established based on selection of Jurkat clones that contained single integrated latent but transcriptionally competent HIV-derived minimal or full length HIV viruses. These HIV-derived latent viruses drive the expression of a reporter gene such as GFP [61,62,63,64], allowing the mechanistic characterization of latency establishment and re-activation. Because they are amenable to large scale biochemical analysis and screens, the latent HIV cell line models have enabled the molecular examination of HIV latency and transcriptional re-activation. Studies using these models and have led to the identification and characterization of not only transcription regulatory factors and signaling pathways involved in HIV latency but also of potential therapeutic molecules for re-activation. However, these are immortalized T cell clones, harboring latent HIV whose activity is susceptible to genomic position and clonal effects of the uniformly integrated latent provirus. Therefore studies using cell line models of HIV latency have limitations as they are not representative of the *in vivo* reservoir of resting memory CD4 T cells harboring a small pool of heterogeneous latent HIV integrations.

Primary cell models have been recently generated in which HIV latency is established in CD4 T cells derived from healthy donors. In the natural host, establishment of HIV latency is thought to occur via two alternative mechanisms: via the transition to the resting state of a cell that was infected with HIV in the active state but becomes quiescent in the process of memory cell generation, or via the direct infection of a resting cell [43]. Currently available primary models of HIV latency attempt to recapitulate these two processes *in vitro* and therefore can be divided in two groups according to the process that is mimicked by the protocol. To reproduce the establishment of latency during transition of an active CD4 T cell to a resting state, cells are infected in the active state and then kept in culture to allow the reversion to the resting state of a subset of the infected population while the activated infected cells die due to apoptosis. The use of defective viruses or antiviral compounds limits the infection to a single replication cycle. This group of protocols includes the Bosque and Planelles method, which results in the establishment of latency in a subset of cells phenotypically similar to the central memory CD4 T cells, which play a major role in the long term maintenance of HIV latency [65]. Conceptually similar methods have increased the efficiency of latently infected cells generation by extending the life span of the cultures through the exogenous expression of the antiapoptotic protein Bcl-2, which promotes the resistance of CD4 cells to apoptosis [66], or trough co-culturing infected cells with a feeder cell line [56,67].

In the second group of protocols, latency is generated in cells infected without prior activation [68,69,70,71]. Although this approach results in the production of latent HIV infected Transitional and Central memory T cells, the main cell types containing latent HIV, infection using in this method is very inefficient and HIV integration infrequent.

The establishment of primary cell models of HIV latency has been very important for HIV eradication studies, particularly for the testing and characterization of putative therapeutic molecules capable of activating latent HIV. However, a recent study showed that there are significant differences in the responses of different models to latency reversing agents (LRAs). This difference is likely due to the fact that each model is representative of only a subset of the *in vivo* target CD4 sub-populations harboring HIV and a restricted number of pathways that contribute to the generation of latency [72].

Confirmation using *ex vivo* monitoring of HIV reactivation in cells derived from patients is therefore required in order to assess the efficacy of LRAs. *Ex vivo* activation of latent HIV in infected patient cells represents the most relevant and powerful system for testing of putative therapeutic molecules and rational drug design. This method is based on the purification of high numbers of resting CD4 T cells from stably suppressed patients followed by treatment with various candidate LRAs [3,5,73,74]. Since the patients enrolled in these studies have no signs of viral replication, it is accepted that HIV present in these cells is in a latent or defective form. However, the very low numbers of CD4 T cells estimated to contain replication competent latent HIV (1:100,000–1:1,000,000)—represent a serious technical hurdle for quantification of HIV re-activation [75]. Viral reactivation can be detected either by quantification of the HIV cellular associated RNA or by co-culturing the cells with uninfected targets in order to amplify and detect re-activated virus. Detection of cellular associated RNA by means of nested or digital droplet PCR is relatively easy to implement and has been proven to be very sensitive in detecting the presence of HIV transcripts [76]. However, it poses some technical challenges such as the necessity to distinguish between read through and LTR-driven transcripts and it does not assess actual virus production [77]. As an alternative, HIV reactivation can be detected via co-culture assays which, although less sensitive and less quantitative, is able to demonstrate actual production of infective virions, which is considered the final goal of reactivation strategies [78,79].

A quantitative co-culture assay together with the single copy assay for the detection of viral RNA in plasma represent the current gold standard methods for measuring HIV reactivation and viral reservoir size in clinical trials [80,81,82]. However, both assays present some limitations mainly related to their suboptimal sensitivity [83,84]. Given the pivotal role that these assays will have in determining the overall efficacy of shock and kill therapies in *in vivo* studies, the development of more accurate and manageable assays is greatly needed.

## 5. Cellular Targets of Latency-Reversal Therapeutics

Our understanding of the key signaling pathways regulating HIV transcription has provided new insights into mechanisms that can be targeted to induce HIV replication and purge the latent reservoir [85]. Initial efforts to induce HIV activation from latency were focused on the use of cytokines and other mitogenic stimuli [86,87]. However, cytokine treatment can lead to a general state of activation causing deterioration of the immune response of the patient, and/or stimulate homeostatic proliferation, thereby increasing the overall number of infected CD4 T cells [11,88].

To date, molecules under evaluation include modulators of chromatin remodeling processes and molecules able to increase the pool of available 5' HIV LTR activators and co-activators. In the following paragraphs will provide an overview of the molecules currently under investigation and their targeted pathways.

### 5.1. The PKC Pathway

One of the most critical pathways in activation of the HIV promoter involves PKC proteins, a family of serine-threonine kinases that is activated in response to TCR stimulation in CD4 T cells [89,90].

The transcription factors NF-κB, NFAT and AP-1 are downstream molecular effectors of the PKC pathway and multiple binding sites for these sequence specific transcription factors are present within the HIV promoter. Under basal conditions, the NF-κB binding sites on the HIV promoter are bound to the inactive homodimeric form of NF-κB, p50/p50, and the active form of the transcription factor, the heterodimer p65/p50, is sequestered in the cytoplasm. Upon PKC activation, the p65/p50 heterodimer is translocated to the nucleus where it replaces the inactive dimers [91]. NFAT, which binds the same sites, is also translocated in the nucleus as a result of calcium/calcineurine induction following PKC activation [92]. Both active NF-κB and NFAT recruit histone acetyltransferases, such as p300/CBP, resulting in acetylation of histone tails and opening of the LTR chromatin structure [93,94].

Different classes of PKC agonists, including phorbol esters, diacylglycerol and ingenols, are able to activate HIV in cellular models of latency and in primary cells from HIV patients. However, given the number of cellular events in which PKC is involved, the majority of these molecules also induces cell proliferation and immune activation. The phorbol ester prostratin and the macrolactone bryostatin-1 have emerged as candidates for HIV activation as they effectively stimulate HIV transcription while showing limited effects on global cell activation [95,96,97,98]. Interestingly, prostratin treatment was shown to also upregulate the expression of p-TEFb in resting CD4 T cells [99]. More recently, a series of prostratin analogues have been synthetized with 100 fold increased potency in inducing HIV transcription while showing a moderate increase of the expression of CD4 surface activation markers [100]. A hexanoate derivative of the PKC inducer ingenol, Ing-B, has been shown to upregulate HIV transcription in cells from HIV positive patients under suppressive therapy and to stimulate latent virus reactivation in a primate model of HIV latency [101,102]. AV6 is an additional promising anti-latency molecule identified through a high throughput screen, which activates latent HIV via stimulation of NFAT mediated transcription [103].

### 5.2. The JNK Pathway

In the JNK cascade, the phosphorylation of c-Jun N-terminal kinase (JNK) drives the activation of the oncoprotein c-Jun, which dimerizes to form the activator protein-1 (AP-1) transcription factor. Multiple AP-1 binding sites are present along the HIV genome, both at the 5' LTR promoter and inside the coding region of the pol gene [33,104]. Interestingly, two mutagenic screenings identified AP-1 sites as major players in HIV transcription as the introduction of deletions in these sites increased the likelihood of latent infections and inhibited HIV reactivation even in presence of fully functional NF-κB signaling [105,106].

Triggering TLR signaling induces JNK pathway mediated activation of HIV transcription. Pam3CSK4, a TLR-1/2 agonist, was shown to induce translocation of NF-κB and AP-1 to the nucleus and concomitant HIV activation in absence of T cell activation and proliferation [107]. A more specific activation of the JNK-AP-1 pathway can be achieved following 8-methoxy-6-methylquinolin-4-ol (MMQO) treatment. Interestingly, despite promoting a significant increase in HIV transcription, treatment with this compound inhibits transcription from TCR signaling targets, thus reducing the likelihood of global cell activation [108]. Farnesyl transferases, additional molecules able to activate HIV transcription through a JNK-dependent pathway, have been identified using high throughput screening on latently infected cell lines [109,110].

### 5.3. The Wnt Pathway

After engagement of the Wnt receptors by Wnt ligands [111], the β-catenin destruction complex, which keeps cytoplasmic levels of β-catenin low, is inactivated. As a result, β-catenin accumulates in the cytoplasm, and translocates into the nucleus where is complexes with LEF1, and activates Wnt target genes [111]. The HIV LTR contains several consensus binding elements for LEF1, the downstream molecular effector of the classical Wnt signaling pathway and LEF1 as the founding member of TCF/LEF transcription factors was originally identified via its interaction with the HIV LTR [112]. We have recently shown that activation of the Wnt pathway via natural ligands and small molecule inhibitors in latently infected cells stimulates HIV transcription and protein production [113]. Moreover, inactivation of the β-catenin destruction complex by siRNA mediated depletion of AXIN1 was shown to activate HIV, demonstrating a positive role for LEF1/β-catenin/Wnt signaling in HIV transcription [114]. In contrast, several studies mainly focusing on astrocytes have also described a repressive role for β-catenin/Wnt on HIV transcription and replication [115,116,117,118,119]. Suppression of HIV replication by β-catenin/Wnt was mediated by the activity of TCF4 [115], suggested to overcome the LEF-driven activation during HIV infection in astrocytes, where it could represent a latency driving mechanism [117,118]. In peripheral blood mononuclear cells treatment with Lithium, a small molecule Wnt pathway activator, was shown to inhibit active HIV replication [120].

### 5.4. The AKT Pathway

In Bcl-2 transduced primary CD4 T cells, NF-κB mediated HIV activation can also be achieved after induction of the Akt pathway by Disulfiram treatment [121]. Disulfiram is an inhibitor of acetaldehyde dehydrogenase used to treat chronic alcoholism and reduces the activity of PTEN, a negative regulator of the Akt signaling pathway [122]. Disulfiram administration to HAART treated patients induced a transient increase in plasma viremia. However, 8 weeks after administration the patients showed no reduction in HIV reservoir size [82].

### 5.5. Histone Deacetylases (HDACs)

Histone Acetylation, or the deposition of positively charged acetyl groups by histone acetyl transferases (HATs) on the histone tails results in the opening of chromatin structure, and facilitating transcription. HDACs are enzymes, which remove the positive acetyl charges from histone tails, resulting in the generation of a more closed and rigid chromatin structure, and their activity at the HIV promoter plays a major role in transcriptional repression and in the onset of HIV latency. Recruitment of HDAC1, HDAC2, HDAC3, as well as HDAC4 to the HIV promoter has been shown to be associated with transcriptional repression [123,124]. Multiple DNA-binding complexes have been shown to recruit HDAC activity to the HIV-1 promoter: HDAC1 was shown to be recruited to the Nuc-1 region of the HIV LTR via the cooperative binding of LSF and YY-1 [40,125], as well as the LTR-bound transcription factors c-Myc and Sp1 [126]. The repressive p50/p50 homodimers occupying the NF-κB LTR sequences have also been shown to recruit HDAC1 to the HIV LTR [127]. Finally, a downstream molecular effector of the Notch pathway, C promoter binding factor 1 (CBF-1) also recruits HDACs to the latent HIV LTR [42,56]. Depletion of YY1, but not c-MYC, in Jurkat cells activated transcription from the HIV promoter. However, depletion of these two transcription factors was not sufficient to disrupt the binding of HDAC complexes to the HIV LTR in Jurkat cells, suggesting that multiple overlapping pathways participate in tethering HDACs to the latent HIV LTR [125].

Because of their role in repressing transcription at the HIV LTR, HDACs are an important target in pharmacological approaches to de-repress and activate latent HIV. HDAC inhibitors (HDACis) are a family of molecules already used in clinical practice that facilitate transcription by inhibiting the activity of HDACs. Indeed, HDAC inhibition was shown to disrupt Nuc-1 and increase HIV transcription [79,128,129,130]. Valproic acid (VPA), an HDACi used in epilepsy and bipolar disorders, was the first compound tested in the context of shock and kill strategies in chronic patients. In the first proof of concept study, Lehrman *et al*. showed a significant decrease in the size of reservoirs after VPA treatment coupled to HAART intensification, however further clinical studies failed to reproduce this finding [131,132,133]. Recently, Vorinostat, Givinostat, Droxinostat, Panobinostat, Romidepsin and Entinostat showed stronger *in vitro* activation properties compared to VPA in latently infected cell lines and in *ex vivo* latency models [134,135,136]. Notably, considerable differences in the efficacy of these compounds in reverting HIV latency may be observed among different cellular models, probably due to differences in the abundance of the specific HDAC isoform inhibited by each compound [137]. Importantly, a well-tolerated dose of Vorinostat (suberoylanilide hydroxamic acid/SAHA) was shown to induce HIV transcription from latently infected cells obtained from HAART treated patients [29]. HDACi-mediated induction of intracellular HIV transcription was confirmed in clinical studies evaluating the effect of multiple daily doses of Vorinostat and thrice-weekly, every-other-week dosing of Panobinostat [138,139]. However, low-level transient increase of viremia was observed only after Panobinostat administration [81]. It is worth noting that viremia is generated by reactivation of intact viruses and that the majority of proviruses found in vivo is defective and thus cannot be effectively targeted by latency reversal treatments. In addition, Ho *et al.* recently showed that a consistent proportion of replication competent proviruses is refractory to latency reversal [75]. A clinical study in which HIV patients were treated with Vorinostat according to the therapeutical scheme in use for cancer patients, showed that Vorinostat treatment induced increase in HIV cellular associated RNA only after the first exposure [80]. Compensatory mechanisms regulating chromatin acetylation are likely at play and may be responsible for decreasing the subsequent response to additional doses during the 24-h dosing intervals tested.

HDAC inhibition promotes activation of different HIV subtypes without inducing massive T cell activation. Therefore, inhibition of these complexes is an attractive approach in re-activation efforts. However, a number of cellular processes depends on histone acetylation and the use of HDACis needs extensive testing in order to minimize unwanted pleiotropic effects [140,141].

### 5.6. Histone Methyl Transferases (HMTs)

Nucleosomal structure of the silenced LTR is characterized by additional markers of condensed heterochromatin such as histone methylation. In particular, methylation of Histone 3 (H3) at position 9 and 27 is generally associated with transcriptional repression [74] and histone methylation marks have been shown to be associated with HIV transcriptional silencing in different latency models [142,143]. Consistently, the HMTs G9a and SUV39H1, the two enzymes that catalize di- and tri-methylation of H3K9 have been shown to physically associate with the latent LTR and mediate repression in latently infected cells from HIV patients [143,144,145]. EZH2, another HMT, is an integral subunit of the Polycomb group repressive complex 2 (PRC2) and catalyzes H3K27 trimethylation of histones. EZH2 was found associated with the latent HIV promoter [142] and is thought to mediate a chromatin environment for sequential docking of other repressive complexes including HDACs, HMTs, DNA methyltransferases and PRC1.

As major players in the epigenetic silencing of the latent HIV promoter, HMTs provide attractive targets for inhibition in HIV re-activation and as a consequence, HMTi’s have been tested as therapeutic inducers of HIV transcription. Thus far, inhibitors targeting the activity of Suv39H1 and G9a, have been tested for HIV re-activation potential. Chaetocin, a specific inhibitor of Suv39H1, and the G9a inhibitor BIX-01294 were both found to induce chromatin relaxation causing HIV reactivation in resting memory CD4 T cells from HAART-treated patients [146,147]. Despite these promising results, due to toxicity and their side effects, these compounds cannot be safely administrated to humans and further studies are needed to identify additional safer HMTi’s for potential therapeutic use.

### 5.7. DNA Methyltransferases

In a screening for the identification of cellular factors involved in HIV latency, Kauder *et al*. identified the methyl-CpG-binding domain protein (MBD2) as a transcriptional repressor. Further investigations determined that MBD2 was associated with the CpG islands flanking the transcription start site and repressed HIV transcription via the recruitment of the NURD complex [148]. Consistent to this hypothesis, treatment of latently infected cells with the methylation inhibitor 5-aza-2'-deoxycytidine (aza-CdR) compromised repression at the HIV promoter [148]. Although not able to activate when used alone, aza-CdR significantly enhanced TNF-α-mediated induction of HIV transcription [149]. Cytosine methylation of the HIV promoter has been observed in both cell line models of latency and in primary cells, and while not necessary for establishment of latency, viruses harboring heavily methylated promoters showed less propensity to reactivation [150]. A significant increase in methylation was observed in HIV promoters isolated from aviremic than viremic patients. However DNA methylation was rarely detected in CD4 T cells obtained from c-ART treated patients [150,151]. Thus, the relevance of this modification to HIV latency remains unclear.

### 5.8. ATP-Dependent Chromatin Remodelers (BAF and CHD3)

The CHD3 and MBD2 containing ATP-dependent chromatin remodeling complex, NuRD has been shown to physically interact with and repress the HIV LTR [152]. We have shown that BAF, a member of the SWI/SNF family of ATP dependent chromatin remodelers, actively positions the repressive HIV LTR Nuc-1 over energetically sub-optimal DNA sequences, leading to the establishment and maintenance of HIV latency [153]. In latently infected cell lines, siRNA mediated knock down of BAF specific subunits leads to HIV activation, identifying the complex as a possible target for reactivation strategies [153,154,155]. INI-1, a core subunit of SWI/SNF was also shown to repress basal HIV promoter activity [156]. Thus, the ATP-dependent chromatin remodeling complexes BAF and NuRD present attractive candidates for therapeutic inhibition in HIV re-activation.

Recently, a spectrum of small molecule inhibitors of the BAF complex have been identified from a screen for the ability to induce expression of bmi1, a specific BAF target gene in mouse embryonic stem cells [157]. Due to their high target specificity, these compounds may present a promising group of molecules aimed at promoting HIV reactivation.

### 5.9. Bromo-Domain-Containing (BET) Factors

RNA pol II pausing is a general regulatory mechanism in eukaryotic cells, and the recruitment of P-TEFb to cellular genes is mediated by BET proteins, in particular Brd4 [158,159]. Interestingly, in the context of HIV expression, Brd4 acts as a repressor, since it directly competes with Tat for binding to P-TEFb, reducing the pool of SEC that would be redirected to HIV transcripts [160]. ShRNA mediated inhibition of BET proteins results in potent activation of HIV transcription [161]. Interestingly, BET inhibition also triggers activation of latent clones that do not express Tat, suggesting that BET proteins are also involved in HIV repression and contribute to maintenance of latency via Tat-independent mechanisms [162]. To explain the Tat-independent BET-mediated modulation of HIV latency, Bohem *et al.* proposed the involvement of a second BET protein in the regulation of HIV latency, Brd2. Similar to Brd4, knock down of Brd2 strongly enhances HIV transcription [162]. Brd2 may directly associate with chromatin remodeling complexes and guide the docking of other inhibitory complexes to the latent HIV promoter. Consistent with the HIV inhibitory role of BETs, the small molecule BET inhibitors JQ1 and IBET-151, has been shown to effectively reactivate HIV from latency in cultured cells and primary T-cell models of latency [163,164,165].

### 5.10. HEXIM

Low levels of active P-TEFb are one of the major barriers that prevent HIV replication in resting CD4 T cells. Inactivation of P-TEFb is dependent on the binding of CyclinT1 and CDK9 to HEXIM1, an inhibitory molecule which is part of the 7SK snRNP [53]. Inhibition of HEXIM using hexamethylene bisacetammide (HMBA) was found to increase the activity of P-TEFb in latently infected cell lines [166]. However due to its poor bioavailability and limited activity in primary *ex vivo* models of latency, this compound has not yet been tested in clinical trials.

## 6. Combinatorial Approaches to Activate Latent HIV

**Figure 1:**
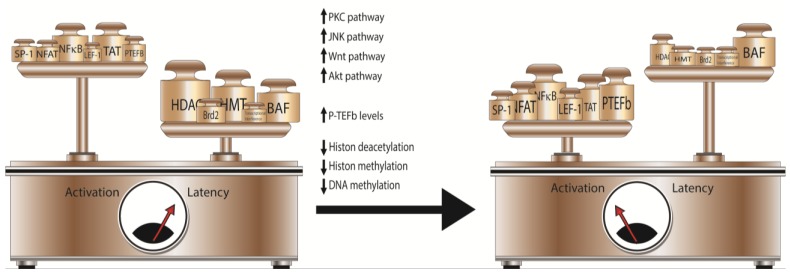
Multiple pathways can be targeted for reverting HIV latency. Silencing of viral transcription results from the concerted activities of LTR-repressive and activating factors and co-factors. The use of combinatorial therapy, targeting multiple pathways involved in the modulation of HIV transcription, will increase the likelihood of reversing the balance from latency to activation.

Because of the complex nature of latency, purging therapies simultaneously targeting multiple mechanisms of latency establishment and maintenance, *i.e.*, combining the inhibition of one or more HIV repressors with one or more inducer of HIV transcription, will likely be the most effective in HIV reactivation (Figure 1). Each silencing event is the unique result of the combination of multiple pathways, and specific characteristics of the integrated provirus, including site of integration, viral subtype and the cell type in which latency is established can hinder HIV reactivation if a single LRA is used.

The majority of latent proviruses from suppressed patients on c-ART is integrated in host genes that are actively transcribed [31,167]. It is widely accepted that latency can derive from integration in actively transcribing genes, through a mechanism called transcriptional interference. Transcriptional interference refers to the inhibition of mRNA synthesis resulting from displacement of transcription factors from the HIV promoter by the host RNA II polymerase [168,169]. However, an extensive analysis of the characteristics of integration sites in *ex vivo* primary models of HIV latency, failed to identify shared integration tracts distinguishing latent from active proviruses [170].

The LTR regions of different HIV subtypes are characterized by specific configurations, mainly resulting from variations of the number and the sequence of transcription binding factors. [106]. As a result, different HIV strains show significant variation in terms of propensity to latency and response to activating stimuli [171,172].

In addition, the specific biological features of each cellular reservoir may play a role in the efficacy of LRAs. Viral reservoirs include different compartments such as the central nervous system, the gut-associated lymphoid tissue and the reproductive tract [12,173]. At the cellular level, latent proviruses can be retrieved in different cell populations: the predominant localization of latent provirus during therapy has been identified in central and transitional memory CD4 T cells [11], although a minimal contribution to HIV reservoir from naive CD 4 T cells has also been reported [16]. The response of HIV promoter to LRAs may differ depending on the specific transcriptional environment within each cell subtype in which latency is established [72].

Finally, it has been recently reported that treating CD4 T cells from completely suppressed patients with strong T cell activators does not induce all replication competent viruses from latently infected cells. Interestingly, some of the clones that are not induced by a single round of stimulation, can be activated subsequently, whereas some remain silent after multiple stimulations [75]. These findings indicate that latency is not only the result of insufficient levels of activation in the latently infected cell, but also depends on stochastic events such as the occupancy of the promoter by cellular activators and the fluctuation of Tat levels [28,174,175]. This stochastic mechanism of transcription activation at the LTR can therefore be exploited by the use of noise enhancers or de-repressors in combination with bona fide activators of the LTR. Recently, in a screening of nearly 1600 FDA approved molecules, Dar *et al.* identified several compounds that modulate basal HIV transcription and synergistically enhance the activity of TNF-α and prostratin [176].

Overall, maintenance of latency depends on a balance between activation and repression of the HIV promoter and targeting multiple pathways at the same time will increase the probability of reaching the threshold that determines HIV activation. Importantly, because of the presence of synergistic effects between individual latency reversal molecules, lower concentrations of each activating compound will be necessary to induce HIV activation. This is an important therapeutic consideration, since at lower concentrations, the toxic side effects and pleiotropic consequence of each molecule on gene expression will be limited. At the same time, because of their synergistic effects on the HIV LTR, combinatorial use of latency reversal molecules can provide a level of specificity for activation of the HIV LTR.

Synergistic activation of HIV transcription following combinatorial treatment with multiple LRAs has been repeatedly reported. Combination of prostratin with Vorinostat [172] as well as with the newly identified HDACi M344 [177] resulted in a synergistic activation of the HIV promoter. Synergistic activation of HIV transcription was reported also after treatment with a different PKC activator, Ing-B, together with the HDACi Vorinostat [178]. Similarly, treatment with Wnt/b-catenin pathway activators or with farnesyl transferase inhibitors synergistically enhanced the effect of Vorinostat [179], and AV6 [103], an NFAT activator, increased the efficacy of valproic acid in activating HIV transcription. These synergistic effects are likely the result of the targeting of different mechanisms: the removal of the repressive chromatin environment by compounds such as HDACi’s, together with the triggering of a transcription activation pathway as would be achieved by AV6. Moreover, HDACi’s can further influence HIV transcription by regulating the acetylation of the viral transactivator Tat and of the cellular transcription factor NF-κB [52,180,181]. Availability of P-TEFB and efficiency of transcriptional elongation represent additional targets for combinatorial therapy. Zhu *et al.* showed that combined treatment with the BET inhibitor JQ1 and prostratin enhanced the reactivation of HIV from latently infected cell lines and, albeit showing more heterogeneous responses, in primary cells [162].

## 7. Conclusions

The knowledge of the multiple mechanisms regulating HIV latency has significantly increased in the last decade, raising hopes for the development of a curative therapy for HIV through shock and kill strategies. Nevertheless, several obstacles remain, which hamper the development of an effective functional cure. Among them, the most immediate obstacle is the development of a therapy, which successfully targets a significant portion of the re-activatable latent reservoir for activation. So far, single molecules tested in preclinical and clinical studies for activation of latent HIV have yielded disappointing results. This supports the notion that a combinatorial cocktail of molecules, which displays synergistic activity and a level of specificity for the HIV promoter will have to be identified and developed. Moreover, a careful evaluation of the second phase of development of a shock-and kill strategy will be necessary. HIV reactivation needs to take place in presence of a fully effective and possibly intensified c-ART able to completely block new rounds of infection and, most importantly, in presence of a functional immune system able to eliminate latently infected cells following reactivation.
